# Credibility of self-reported health parameters in elderly population

**DOI:** 10.1017/S1463423620000201

**Published:** 2020-06-10

**Authors:** Roi Amster, Iris Reychav, Roger McHaney, Lin Zhu, Joseph Azuri

**Affiliations:** 1 Sackler Faculty of Medicine, Tel Aviv University, Tel Aviv, Israel; 2 Department of Industrial Engineering & Management, Ariel University, P.O.B 40700, Ariel, Israel; 3 Daniel D. Burke Chair for Exceptional Faculty, Professor and University Distinguished Teaching Scholar, Management Information Systems, Kansas State University, Manhattan, KS 66506, USA; 4 Maccabi Healthcare Services, Tel Aviv, Israel

**Keywords:** Health Belief Model, self-reporting, blood pressure, tablet, BMI, elderly

## Abstract

**Aim:**

Examining the credibility of self-reported height, weight, and blood pressure by the elderly population using a tablet in a retirement residence, and examining the influence of health beliefs on the self-reporting credibility.

**Background:**

Obesity is a major problem with rising prevalence in the western world. Hypertension is also a significant risk factor for cardiovascular diseases. Self-report, remotely from the clinic, becomes even more essential when patients are encouraged to avoid visiting the clinic as during the COVID-19 pandemic. Self-reporting of height and weight is suspected of leading to underestimation of obesity prevalence in the population; however, it has not been well studied in the elderly population.

The Health Belief Model tries to predict and explain decision making of patients based on the patient’s health beliefs.

**Methods:**

Residents of a retirement home network filled a questionnaire about their health beliefs regarding hypertension and obesity and self-reported their height, weight, and blood pressure. Blood pressure, height, and weight were then measured and compared to the patients’ self-reporting.

**Findings:**

Ninety residents, aged 84.90 ± 5.88, filled the questionnaire. From a clinical perspective, the overall gap between the measured and the self-reported BMI (*M* = 1.43, *SD* = 2.72), which represents an absolute gap of 0.74 kilograms and 2.95 centimeters, is expected to have only a mild influence on the physician’s clinical evaluation of the patient’s medical condition. This can allow the physician to estimate their patient’s BMI status before the medical consultation and physical examination upon the patient’s self-reporting. Patients’ dichotomous (normal/abnormal) self-report of their blood pressure condition was relatively credible: positive predictive value (PPV) of 77.78% for normal blood pressure (BP) and 78.57% for abnormal BP. The relatively high PPV of BP self-reporting demonstrates an option for the physician to recognize patients at risk. Regression analysis found no correlation between the anthropometric parameters and the Health Belief Model.

## Background

Obesity is widespread in the western-world population and causes major medical concerns due to its direct link to increased risk of mortality (Flegal *et al.*, 2005). In addition to mortality, obesity has been demonstrated to correlate with many medical conditions such as diabetes (Mushcab *et al.*, 2017), hypertension, and cardiovascular disease (Flegal *et al.*, 2002). Body mass index (BMI) is a common and effective method to estimate obesity since its components (height and weight) are readily available and easy to measure. The widespread use of BMI enables researchers to compare results in different studies (Flegal *et al.*, 2013). Particularly among older population members, very high or very low BMI correlates with higher mortality (Miller and Wolfe, 2008) and therefore becomes an indicator that an individual needs to be under close medical observation. Hypertension is the third most influential mortality factor (Ezzati *et al.*, 2002) and is responsible for at least 45% of deaths due to heart disease and more than 50% of deaths due to stroke (WHO, 2013). Hypertension prevalence rises in the older population, resulting in higher levels of treatment within this group (Ong *et al.*, 2007). Blood pressure measurement is the primary tool for diagnosis, management, treatment, and research of hypertension (O’Brien *et al*., 2005).

Self-reported data for height and weight is commonly used, but can lead to underestimating obesity since people tend to report greater heights, and lower weights than actual measurements indicate (Ahima and Lazar, 2013; Hattori and Sturm, 2013). The credibility of self-reported height and weight can be estimated by the gap between self-reported data and measured data. Self-reporting of blood pressure has also been researched, but the evidence is inconsistent concerning its overall credibility (Goldman *et al.*, 2003; Chun *et al.*, 2016). The credibility of self-reported BP can be estimated by the gap between self-reported data and measured data or by comparing patients’ dichotomous (normal\abnormal) self-report of their blood pressure to their actual BP measurement.

As life expectancy keeps rising, and the percentage of elderly, aged 65 and older (Brody *et al.*, 1987; Kaye *et al.*, 2010), is expected to increase, medical systems will need to treat more and more older patients who require close monitoring and engage in self-management practices (Morgan *et al*., 2017). Past studies have shown that the lack of time takes a toll on the ability of physicians to treat their patients (Yarnall *et al.*, 2003). Self-reporting using digital aids can be another tool in the physician’s arsenal that could help monitor patients (Linderholm *et al.*, 2019; Pereira *et al.*, 2019). Self-reporting, while practicing telemedicine, becomes even more essential when patients are encouraged to avoid visiting the clinic as during the COVID-19 pandemic (Hollander and Carr, 2020). However, this is only useful if self-report credibility can be improved.

The Health Belief Model (HBM) predicts and explains patient decision making related to medical issues (Jones *et al*., 2015), for instance, in the context of diabetes (Gillibrand and Stevenson, 2006) or breast cancer (Norman and Brain, 2005). The model suggests patients’ decisions correspond to her or his evaluation of the four following factors: perceived benefits, perceived barriers, perceived severity, and perceived susceptibility (Rosenstock, 1966, 1974).

In general, the model’s ability to predict and explain patterns of behavior has been proven many times (Janz and Becker, 1984). In particular, the HBM model succeeded in predicting medical decision approaches among older population members (Rundall and Wheeler, 1979; Esmaelzadeh *et al.*, 2018) and others (Shafer *et al.*, 2018). However, this model was not used to investigate its effectiveness in predicting self-reported health parameters’ credibility.

Our goal in this study is to examine the credibility of self-reported height, weight, and blood pressure by the elderly population living in a retirement residence to a tablet and explore the influence of health beliefs on the self-reporting credibility.

## Methods

### Population and sample

The study was conducted in a network of three retirement residences. All residents are at least 65 years old. All subjects who volunteered for the study were 70–97 years old, ambulatory, and without any significant cognitive impairment. The residents were invited to participate in the study through advertising inside the retirement residences. The sample size was estimated to be at least 85 participants using Winpepi software (Abramson, 2004), based on 5% significance level and 80% power level and a *t*-test for correlation between the anthropometric variables and HBM score with a clinical significance of *r* = 0.30.

### Questionnaire

The questionnaire (Appendix 1) was based on questions derived from prior studies that dealt with the relation between HBM and obesity or hypertension (Weissfeld *et al.*, 1990; Desmond *et al.*, 1992; Park, 2011; Scotland, 2012). The questions were scored on a 1–5 scale (strongly agree–strongly disagree). The questions were translated and proofread after adaptation to the study population.

The study’s demographic questions included age, gender, education, religious status, hospitalization, and frequency of visiting doctors and nurses in the past year. At the end of the questionnaire, the participants were asked to self-report height, weight, and blood pressure to the best of their knowledge, and to grade their blood pressure as low, normal, or high.

The questionnaire was first executed as a pilot to five participants over the age of 65 years that visited a primary medical clinic. Minor changes to wording resulted.

### Data collection

Each study day, residents living in “Bait Balev” retirement residences network were invited to participate in the study. The residents were asked to fill in a digital version of the questionnaire on a tablet. Finally, a team member measured and recorded the participant’s actual parameters. All data were kept anonymous.

Blood pressure measurements were completed according to the Joint National Committee (JNC) recommendations (Chobanian *et al*., 2003). Each participant was measured three times, and the average of the second and third times was recorded. Two of the 90 participants refused to complete the third measurement, and their second measurement was recorded. Systolic blood pressure (SBP) higher than 160 or diastolic blood pressure (DBP) higher than 100 was immediately reported to the medical staff in the retirement residence for further consideration of medical actions.

### Data analysis

In the current study, we used three standard adult-elderly population thresholds for systolic blood pressure hypertension (Mancia *et al*., 2009): SBP < 140 – valid for all adult population; SBP > 160 – begin treatment in elderly (>80 yrs); SBP = 140–150 target pressure for elderly (>80 yrs).

Measured and reported BMI were calculated by weight (in kilograms)/height (in meters)^2^ that were measured or reported, respectively. BMI data were analyzed and broken into categories described as underweight (BMI below 18.5), normal (BMI 18.5–24.9), overweight (BMI 25.0–29.9), and obese (BMI above 30.0). BMI and SBP gaps were defined as the difference between the reported to the measured BMI and SBP, respectively.

Data were presented using descriptive statistics and analyzed by Student *t*-test and regression model.

All data analyses were completed using SPSS software.

### Ethics committee approval

The study was approved by the Bait Balev local ethics committee (IRB) before commencing the trial (BBL-0063-18). A waiver was given for written informed consent from patients as all the questionnaires were fully anonymized.

## Results

Between May and August 2018, 90 residents participated in the study. Only two residents that we approached refused to participate. Table 1 shows the demographic and general characteristics of the participants, and Table 2 summarizes the measured and self-reported BMI and BP.

**Table 1.** Demographic and general characteristics of participants

Characteristics	*N* (90)	% (100)	Mean	Standard deviation
**Gender**				
Female	74	82.2		
Male	16	17.8		
**Age**			84.9	5.88
70–75	2	2.2		
75–80	16	17.8		
80–85	26	28.9		
85–90	23	25.6		
90–95	21	23.3		
95+	2	2.2		
**No. visits to primary care physician in the last 3 months**	89*		1.69	1.67
0 times	26	28.8		
1–3 times	50	55.6		
4 or more times	14	15.6		
**No. of visits to a nurse in the last 3 months**			10.5	25.6
0 times	35	38.8		
1–3 times	32	35.6		
4 or more times	23	25.6		
**Hospitalization in last year**				
Yes	12	13.3		
No	78	86.7		
**Education**				
High school without matriculation examination	34	37.8		
High school with matriculation examination	24	26.7		
Higher academic education	32	35.5		
**Religious status**				
Secular	68	75.6		
Traditional	22	24.4		

a One person who visited the doctor 45 times in the last 90 days was excluded from the sample.

**Table 2.** Self-report, actual measurements and gap of height, weight and body mass index

	*N*	Mean	SD	Min	Q1	Median	Q3	Max
Self-reported weight	89	68.07	12.99	45.00	60.00	66.00	76.00	100.00
Self-reported height	89	158.82	8.61	140.00	152.00	158.00	165.00	180.00
Self-reported BMI	89	26.92	4.30	17.26	23.53	26.64	29.64	39.03
Measured weight	89	68.81	13.04	46.20	59.10	67.10	77.80	100.00
Measured height	89	155.87	8.10	132.00	150.00	156.00	160.00	179.00
Measured BMI	89	28.35	5.15	18.05	24.65	27.82	31.54	47.41
Weight gap	89	0.74	3.98	−10.20	−0.40	1.00	2.50	20.00
Height gap	89	−2.95	5.64	−21.00	−6.00	−2.00	0.00	15.00
BMI gap	89	1.43	2.72	−6.68	0.17	1.28	2.65	10.67

BMI – Body Mass Index.

Self-reported BMI refers to the calculation of the self-reported measurements.

We excluded one self-reported height of a 162 cm woman who said she doesn’t know her height, but because it was mandatory to self-report she wrote she is 115 cm.

The categorized calculated self-reported BMI (underweight 1.14%, normal 34.09%, overweight 44.32%, and obese 20.45%) vs. the measured BMI (1.14%, 28.41%, 31.82%, and 38.63%, respectively) were lower for the obese group and higher for the normal and overweight groups.

Overall, 45.55% of the study participants self-reported their blood pressure while 54.45% did not know their actual blood pressure levels and therefore did not report it. Of those responding, 80.00% of the participants self-reported that their blood pressure was normal, 15.56% self-reported that it was high, none reported low, and 4.44% self-reported they did not know. Self-reported SBP (*M* = 134.90, *SD* = 15.70, *n* = 41) was lower than the measured SBP (*M* = 150.30, *SD* = 27.20), which led to a SBP gap (*M* = 15.88, *SD* = 27.80). Self-reported DBP (*M* = 73.30, *SD* = 8.02, *n* = 41) was lower than the measured DBP (*M* = 81.20, *SD* = 12.90), which led to the DBP gap (*M* = 7.34, *SD* = 13.10). Measured and self-reported BP were compared according to the self-reported BP Categories (Table 3).

**Table 3.** The relationship between self-reported blood pressure normality to actual blood pressure measurements

	Self-reported blood pressure	*N*	Missing	Mean	*SD*	Min	Q1	Median	Q3	Max
Measured SBP	Normal	72	0	146.93	24.59	104	126.5	146.0	159.5	215
	Abnormal	14	0	168.71	35.80	130	145.0	157.5	181.0	243
	I don’t know	4	0	145.50	15.67	127	132.5	148.0	158.5	159
Measured DBP	Normal	72	0	80.32	12.85	57	70.0	79.0	87.5	122
	Abnormal	14	0	86.29	12.47	72	78.0	82.0	88.0	118
	I don’t know	4	0	78.25	14.06	66	67.0	75.5	89.5	96
Self-reported SBP	Normal	36	36	133.39	14.23	115	120.0	130.0	140.0	170
	Abnormal	5	9	146.00	22.75	110	140.0	150.0	165.0	165
	I don’t know	0	4	non	NA	NA	NA	NA	NA	NA
Self-reported DBP	Normal	36	36	72.14	6.80	60	66.5	70.0	78.0	90
	Abnormal	5	9	81.60	11.84	70	73.0	80.0	85.0	100
	I don’t know	0	4	non	NA	NA	NA	NA	NA	NA

SBM – Systolic Blood Pressure; DBP-Diastolic Blood Pressure.

For participants who reported normal BP (*n* = 72), a measured SBP of <160 was observed in 56 participants (average BP 137.02/77.46), while 16 participants of this group had an actual high pressure (average BP 181.62/90.31). For participants who reported high pressure (*n* = 14), a measured SBP of >140 was observed in 11 participants (average BP 178.63/88.64), while only 3 participants of this group had an actual normal BP (average BP 131.13/77.66).

Significant gap comparisons in BMI and SBP were found, and the analysis by different BMI categories is shown in Table 4.

**Table 4.** Body mass index and systolic blood pressure gaps explained by different body mass index categories

	*N*	% (100)	Mean	*SD*	*p*	*d*	*t*
BMI gap by BMI categories							
Normal-weight BMI gap	24	28.4	−0.026	2.015			
Overweight BMI gap	29	31.8	1.529	1.831			
Obese BMI gap	34	38.7	2.246	3.34			
SBP gap by BMI categories							
Normal-weight SBP gap	11	28.2	5.182	16.582			
Obese SBP gap	17	43.6	24.647	26.65			
BMI gap comparison							
Normal-weight to overweight					.005	−0.81	(47.11) = −2.92
Overweight to obese					.002	−0.79	(54.87) = −3.22
SBP gap comparison							
Normal-weight to obese					.025	−0.84	(25.99) = −2.38

BMI = Body Mass Index; SBM = Systolic Blood Pressure; DBP = Diastolic Blood Pressure.

Data from the questionnaires were used to calculate HBM scores. Eight of the ten different questionnaire categories had a Cronbach’s Alpha higher than 0.60 (Table 5).

**Table 5.** Internal consistency reliability

Characteristics	Cronbach’s Alphas	Number of questions
General HBM	0.647	3
**Hypertension**		
Perceived barriers	0.264	5
Perceived benefits	0.614	6
Perceived susceptibility	0.777	6
Perceived severity	0.796	7
**Obesity**		
Perceived barriers 1	0.672	5
Perceived barriers 2	0.435	5
Perceived benefits	0.910	6
Perceived susceptibility	0.621	4
Perceived severity	0.702	4

### HBM score

We used a regression analysis to test the correlation between height, weight, and BMI gap and the relationship of these items to gender-adjusted HBM scores. The following results were found:

BMI gap – The results of the regression indicated that the seven variables explained 17.50% of the variance of BMI gap (*R* ^2^ = 0.17, *F* (7,81) = 2.19, *p* < .05). Perceived severity significantly predicted BMI gap *β* = −1.32, *t*(81) = 0.43, *p* < .001, which indicated when perceived severity increased, the height gap decreased. Gender was also a significant factor affecting the BMI gap, *β* = 1.67, *t*(81) = 0.74, *p* < .05, which indicated that female self-reports had a higher BMI gap than male’s self-reports by 1.67.

Height gap – the results of the regression indicated that the seven variables explained 16.50% of the variance (*R* ^2^ = 0.16, *F*(7,80)= 2.25, *p* < .05). Perceived severity significantly predicted the height gap, *β* = 2.71, *t*(80) = 0.89, *p* < .01. It indicated when perceived severity increased, the height gap increases.

Weight gap – Perceived Barriers 2 significantly predicted the weight gap, *β* = −1.53, *t*(81) = 0.63, *p* < .05, which indicates when the perceived barriers increases, the weight gap decreased. But the *F*-statistic was insignificant, which suggests the value of the model is very limited on the weight gap.

In a regression that tested the correlation between BP gap and gender-adjusted HBM score, we determined that perceived susceptibility significantly predicted DBP, *β* = −6.67, *t*(34) = 2.95, *p* < .05, which indicated when the participant perceived susceptibility increased, the DBP gap decreased. Furthermore, we ran a logistic regression and analyzed the self-reported BP status and measured BP status. The results were not significant. The chi-square test may have indicated the self-reported data did not have enough power to infer the measured BP. Since *R* ^2^ was relatively low in both cases, even though there were some significant findings, we believe that the HBM score does not explain either the BMI gap or the BP gap. Therefore, generally, we would say that the HBM score was not a good predictor for self-reported data credibility.

Regression analysis was used to test the correlation between height, weight, blood pressure, and BMI gap. The relationships of these items to gender-adjusted HBM score and demographic parameters were not significant.

## Discussion

The elderly population is a unique population that requires closer than average monitoring due to their higher incidence of morbidity and mortality (Kennedy *et al*., 2014), and BP and BMI measurements can alter the medical approach to this population. Self-reporting of blood pressure and anthropometric parameters can assist triage and simplify a remote approach to the patient. As there is still a lack of data in the literature regarding the elderly population, the current study examined the credibility of self-reporting by the elderly population living in a retirement residence. In the older population, in particular, very high or very low BMI correlates with higher mortality (Miller and Wolfe, 2008) and therefore suggests closer monitoring for the patient. From a clinical perspective, the overall BMI gap in the current study (*M* = 1.43, *SD* = 2.72), which represents an absolute gap of 0.74 kilograms and 2.95 centimeters, is expected to have only a mild clinical influence, if any, on the physician’s evaluation of the patient’s medical condition. This can allow the physician to estimate their patient’s BMI status before the medical consultation and examination upon the patient self-reporting.

Previous studies showed self-reported heights to be higher and weights to be lower than the actual measurements (Ahima and Lazar, 2013; Hattori and Sturm, 2013). However, this study shows the anthropometric indices’ credibility to be highest in the normal-weight group (BMI gap: *M* = −0.02, *SD* = 2.01), deteriorate through overweight’s (*M* = 1.52, *SD* = 1.83) and to be worst in the obese group (*M* = 2.24, *SD* = 3.34). Taking into account the actual BMI group levels, these BMI gaps should not make a significant change in the clinical approach to the patient, which makes the height–weight–BMI self-reporting even more credible in the clinical perspective.

Similar to the lower credibility in the BMI self-report, the obese patients were also less credible in BP self-reporting in comparison with the normal-weight group. This could be due to several reasons such as that the individual is not willing to accept their current health risk, society’s general view of obesity, and the pressure that puts on an individual to conform to an `acceptable’ archetype or the individual’s body image as healthy or not. Further studies are needed to address these questions.

In this study, there was a high yield between the participants’ definition of normal vs. high blood pressure and their self-report of blood pressure values (133.38/72.13 vs. 146.00/81.60, respectively).

Target blood pressure for the oldest-old people, aged 85 and older (Rosenwaike, 1985; Rogers, 1999), has long been debated, and inconsistencies regarding the optimal BP for this population exist in different guidelines since there is a lack of clear evidence regarding this issue (Garrison *et al*., 2017; Anker *et al*., 2018). Patients’ dichotomous (normal\abnormal) self-report of their blood pressure condition was relatively credible: positive predictive value of 77.78% for normal BP (SBP < 160) and 78.57% for abnormal BP (SBP > 140). These relatively high PPVs could help physicians identify patients at risk through self-report outside of the medical encounter, e.g., if a patient claims a normal BP, it would be reasonable to assume there is no need for a change in hypertension treatment; while for those who claim a high BP, it warrants a close follow up and BP measurements. Nevertheless, as the PPV is not as accurate as a real measurement, we find this method relevant for monitoring patients from afar, but not as a replacement for actual measurement during the medical encounter.

The HBM was not used previously to investigate its effectiveness in predicting self-reported health parameters’ credibility. In the current study, we also concluded HBM score was not a sufficient predictor for self-report credibility. More research remains to determine if there is better predictability of self-report in other HBM subtypes.

## Conclusions

The gap between measured and self-reported BMI has only a mild influence regarding the evaluation of a patient’s metabolic status. Therefore, we recommend considering the use of self-reporting weight and height using a tablet among the elderly population when direct measurements cannot be taken. COVID-19 pandemic emphasizes the need for the physician to monitor his patients from afar due to the current need to minimize physical encounters. Self-reporting has the potential to be an essential tool in the physician’s toolbox while practicing telemedicine. The relatively high PPV of BP self-reporting demonstrates an option for the physician to recognize patients at risk. Further studies could identify the most credible subjects in this area.

## Limitations

Membership bias could be present because the population who lives in the retirement residence were generally of higher socioeconomic levels than the general population. Another potential bias is volunteering bias because participation was not mandatory; hence participants might be more aware of their medical condition and might possess better than average medical knowledge. However, in the current study, refusal to participate was negligible. Finally, the use of self-reporting to a tablet was also a potential limitation, especially in the elderly population. However, this issue was shown not to be a significant limitation, as the questionnaire was very intuitive and user friendly. Help was given to few participants who required specific instructions on how to use the tablet.

## References

[ref1] Abramson JH (2004). WINPEPI (PEPI-for-Windows): computer programs for epidemiologists. Epidemiologic Perspectives & Innovations 1 (1), 6. 15606913 10.1186/1742-5573-1-6 PMC544871

[ref2] Ahima RS and Lazar MA (2013) The health risk of obesity—better metrics imperative. Science 341 (6148), 856–858. 23970691 10.1126/science.1241244

[ref3] Anker D , Santos-Eggimann B , Santschi V , Del Giovane C , Wolfson C , Streit S , Rodondi N and Chiolero A (2018) Screening and treatment of hypertension in older adults: less is more? Public Health Reviews 39 (1), 26. 30186660 10.1186/s40985-018-0101-z PMC6120092

[ref4] Brody JA , Brock DB and Williams TF (1987) Trends in the health of the elderly population. Annual Review of Public Health 8 (1), 211–234. 10.1146/annurev.pu.08.050187.001235 3555523

[ref5] Chobanian AV , Bakris GL , Black HR , Cushman WC , Green LA , Izzo JL , Jones DW , Materson BJ , Oparil S , Wright JT and Roccella EJ (2003) Seventh report of the joint national committee on prevention, detection, evaluation, and treatment of high blood pressure. Hypertension 42 (6), 1206–1252. 14656957 10.1161/01.HYP.0000107251.49515.c2

[ref6] Chun H , Kim I and Min K (2016) Accuracy of self-reported hypertension, diabetes, and hypercholesterolemia: analysis of a representative sample of Korean older adults. Osong Public Health and Research Perspectives 7 (2), 108–115. 27169009 10.1016/j.phrp.2015.12.002 PMC4850372

[ref7] Desmond SM , Price JH , Roberts SM and Pituch MJ (1992) Perceptions of hypertension in black and white adolescents. Health Values: The Journal of Health Behavior, Education & Promotion 16 (2), 3–10.

[ref8] Esmaelzadeh S , Salehi L and Esmaelpour R (2018) An investigation of self-medication and its correlates among community dwelling elderly population by applying health belief model. Nursing Practice Today 5 (3), 318–325.

[ref9] Ezzati M , Lopez AD , Rodgers A , Vander Hoorn S and Murray CJ (2002) Comparative Risk Assessment Collaborating Group. Selected major risk factors and global and regional burden of disease. The Lancet 360 (9343), 1347–1360. 10.1016/S0140-6736(02)11403-6 12423980

[ref10] Flegal KM , Carroll MD , Ogden CL and Johnson CL (2002) Prevalence and trends in obesity among US adults, 1999–2000. JAMA 288 (14), 1723–1727. 12365955 10.1001/jama.288.14.1723

[ref11] Flegal KM , Graubard BI , Williamson DF and Gail MH (2005) Excess deaths associated with underweight, overweight, and obesity. JAMA 293 (15), 1861–1867. 15840860 10.1001/jama.293.15.1861

[ref12] Flegal KM , Kit BK , Orpana H and Graubard BI (2013) Association of all-cause mortality with overweight and obesity using standard body mass index categories: a systematic review and meta-analysis. JAMA 309 (1), 71–82. 23280227 10.1001/jama.2012.113905 PMC4855514

[ref13] Garrison SR , Kolber MR , Korownyk CS , McCracken RK , Heran BS and Allan GM (2017) Blood pressure targets for hypertension in older adults. Cochrane Database of Systematic Reviews (8), 1465–1858. 10.1002/14651858.CD011575.pub2 PMC6483478 28787537

[ref14] Gillibrand, R. , and Stevenson, J. (2006). The extended health belief model applied to the experience of diabetes in young people. British Journal of Health Psychology, 11(1), 155–169. 16480561 10.1348/135910705X39485

[ref15] Goldman N , Lin I , Weinstein M and Lin Y (2003) Evaluating the quality of self-reports of hypertension and diabetes. Journal of Clinical Epidemiology 56 (2), 148–154. 12654409 10.1016/s0895-4356(02)00580-2

[ref16] Hattori A and Sturm R (2013) The obesity epidemic and changes in self-report biases in BMI. Obesity 21 (4), 856–860. 23712990 10.1002/oby.20313 PMC5800501

[ref17] Hollander JE and Carr BG (2020) Virtually perfect? Telemedicine for covid-19. New England Journal of Medicine [published online ahead of print, 2020 Mar 11]. doi:10.1056/NEJMp2003539. 32160451

[ref18] Janz NK and Becker MH (1984) The health belief model: a decade later. Health Education Quarterly 11 (1), 1–47. 6392204 10.1177/109019818401100101

[ref19] Jones CL , Jensen JD , Scherr CL , Brown NR , Christy K and Weaver J (2015) The health belief model as an explanatory framework in communication research: exploring parallel, serial, and moderated mediation. Health Communication 30 (6), 566–576. 25010519 10.1080/10410236.2013.873363 PMC4530978

[ref20] Kaye AD , Baluch A and Scott JT (2010) Pain management in the elderly population: a review. Ochsner Journal 10 (3), 179–187. 21603375 PMC3096211

[ref21] Kennedy BK , Berger SL , Brunet A , Campisi J , Cuervo AM , Epel ES , Franceschi C , Lithgow GJ , Morimoto RI , Pessin JE , Rando TA , Richardson A , Schadt EE , Wyss-Coray T and Sierra F (2014) Geroscience: linking aging to chronic disease. Cell 159 (4), 709–713. 25417146 10.1016/j.cell.2014.10.039 PMC4852871

[ref22] Linderholm M , Törnvall E , Yngman-Uhlin P and Hjelm K (2019) Self-rated health, lifestyle habits and risk assessment in 75-year-old persons attending preventive clinic visits with a nurse in primary health care: a cross-sectional study. Primary Health Care Research & Development 20 (e88), 1–10. 10.1017/S1463423619000136 PMC6609977 32799984

[ref23] Mancia G , Laurent S , Agabiti-Rosei E , Ambrosioni E , Burnier M , Caulfield MJ , Cifkova R , Clement D , Coca A , Dominiczak A , Erdine S , Fagard R , Farsang C , Grassi G , Haller H , Heagerty A , Kjeldsen SE , Kiowski W , Mallion JM , Manolis A , Narkiewicz K , Nilsson P , Olsen MH , Rahn KH , Redon J , Rodicio J , Ruilope L , Schmieder RE , Struijker-Boudier HA , Van Zwieten PA , Viigimaa M and Zanchetti A (2009) Reappraisal of European guidelines on hypertension management: a European Society of Hypertension Task Force document. Blood Pressure 18 (6), 308–347. 20001654 10.3109/08037050903450468

[ref24] Miller SL and Wolfe RR (2008) The danger of weight loss in the elderly. Journal of Nutrition, Health & Aging 12 (7), 487–491.10.1007/BF0298271018615231

[ref25] Morgan HM , Entwistle VA , Cribb A , Christmas S , Owens J , Skea ZC and Watt IS (2017) We need to talk about purpose: a critical interpretive synthesis of health and social care professionals’ approaches to self-management support for people with long-term conditions. Health Expectations 20 (2), 243–259. 27075246 10.1111/hex.12453 PMC5354019

[ref26] Mushcab H , Kernohan WG , Wallace J , Harper R and Martin S (2017) Self-management of diabetes mellitus with remote monitoring: a retrospective review of 214 cases. International Journal of E-Health and Medical Communications (IJEHMC) 8 (1), 52–61.

[ref27] Norman P and Brain K (2005) An application of an extended health belief model to the prediction of breast self-examination among women with a family history of breast cancer. British Journal of Health Psychology 10 (1), 1–16. 15826330 10.1348/135910704X24752

[ref28] O’Brien E , Asmar R , Beilin L , Imai Y , Mancia G , Mengden T , Myers M , Padfield P , Palatini P , Parati G , Pickering T , Redon J , Staessen J , Stergiou G and Verdecchia P (2005). Practice guidelines of the European Society of Hypertension for clinic, ambulatory and self blood pressure measurement. Journal of Hypertension 23 (4), 697–701. 15775768 10.1097/01.hjh.0000163132.84890.c4

[ref29] Ong KL , Cheung BM , Man YB , Lau CP and Lam KS (2007) Prevalence, awareness, treatment, and control of hypertension among United States adults 1999–2004. Hypertension 49 (1), 69–75. 17159087 10.1161/01.HYP.0000252676.46043.18

[ref30] Park D (2011) Utilizing the health belief model to predicting female middle school students’ behavioral intention of weight reduction by weight status. Nutrition Research and Practice 5 (4), 337–348. 21994529 10.4162/nrp.2011.5.4.337 PMC3180685

[ref31] Pereira MG , Pedras S and Ferreira G (2019). Self-reported adherence to foot care in type 2 diabetes patients: do illness representations and distress matter? Primary Health Care Research & Development 20 (e40), 1–8. 10.1017/S1463423618000531 PMC6536758 30095065

[ref32] Rogers CC (1999) Growth of the oldest old population and future implications for rural areas. Rural America/Rural Development Perspectives 14 (2221-2019-2678), 22–26.

[ref33] Rosenstock I (1966) Why people use health services. Why people use health services. The Millbank Memorial Fund Quarterly 44, 94–127. 5967464

[ref34] Rosenstock IM (1974) Historical origins of the health belief model. Health Education Monographs 2 (4), 328–335. 10.1177/109019817800600406 299611

[ref35] Rosenwaike I (1985) A demographic portrait of the oldest old. The Milbank Memorial Fund Quarterly. Health and Society, 187–205. 3846809

[ref36] Rundall TG and Wheeler JR (1979) Factors associated with utilization of the swine flu vaccination program among senior citizens in Tompkins County. Medical Care 17 (2), 191–200. 215853 10.1097/00005650-197902000-00009

[ref37] Scotland AM (2012) Health beliefs and knowledge related to management of hypertension among adult dominicans. Miami Shores, FL: Barry University School of Nursing.

[ref38] Shafer A , Kaufhold K and Luo Y (2018) Applying the health belief model and an integrated behavioral model to promote breast tissue donation among Asian Americans. Health Communication 33 (7), 833–841. 28467235 10.1080/10410236.2017.1315678

[ref39] Weissfeld JL , Kirscht JP and Brock BM (1990) Health beliefs in a population: the Michigan blood pressure survey. Health Education Quarterly 17 (2), 141–155. 2347692 10.1177/109019819001700202

[ref40] World Health Organization. (2013). A Global Brief On Hypertension: Silent Killer, Global Public Health Crisis: World Health Day 2013 (No. WHO/DCO/WHD/2013.2). World Health Organization.

[ref41] Yarnall KS , Pollak KI , Østbye T , Krause KM and Michener JL (2003). Primary care: is there enough time for prevention? American Journal of Public Health 93 (4), 635–641. 12660210 10.2105/ajph.93.4.635 PMC1447803

